# Operando monitoring transition dynamics of responsive polymer using optofluidic microcavities

**DOI:** 10.1038/s41377-021-00570-1

**Published:** 2021-06-16

**Authors:** Da-Quan Yang, Jin-hui Chen, Qi-Tao Cao, Bing Duan, Hao-Jing Chen, Xiao-Chong Yu, Yun-Feng Xiao

**Affiliations:** 1 School of Information and Communication Engineering, State Key Laboratory of Information Photonics and Optical Communications, Beijing University of Posts and Telecommunications, Beijing, 100876 China; 2 Institute of Electromagnetics and Acoustics, Xiamen University, Xiamen, 361005 China; 3 State Key Laboratory for Mesoscopic Physics and Frontiers Science Center for Nano-optoelectronics, School of Physics, Peking University, Beijing, 100871 China; 4 Department of Physics and Applied Optics Beijing Area Major Laboratory, Beijing Normal University, Beijing, 100875 China; 5 Collaborative Innovation Center of Extreme Optics, Shanxi University, Taiyuan, 030006 China; 6 Peking University Yangtze Delta Institute of Optoelectronics, Nantong, 226010 China

**Keywords:** Optofluidics, Optical sensors, Microresonators

## Abstract

Optical microcavities have become an attractive platform for precision measurement with merits of ultrahigh sensitivity, miniature footprint and fast response. Despite the achievements of ultrasensitive detection, optical microcavities still face significant challenges in the measurement of biochemical and physical processes with complex dynamics, especially when multiple effects are present. Here we demonstrate operando monitoring of the transition dynamics of a phase-change material via a self-referencing optofluidic microcavity. We use a pair of cavity modes to precisely decouple the refractive index and temperature information of the analyte during the phase-transition process. Through real-time measurements, we reveal the detailed hysteresis behaviors of refractive index during the irreversible phase transitions between hydrophilic and hydrophobic states. We further extract the phase-transition threshold by analyzing the steady-state refractive index change at various power levels. Our technology could be further extended to other materials and provide great opportunities for exploring on-demand dynamic biochemical processes.

## Abstract (web summary)

Operando monitoring transition dynamics of responsive polymer via a self-referencing optofluidic microcavity is demonstrated. The refractive index and temperature information of the analyte during the phase-transition process are precisely decoupled.

## Graphical abstract

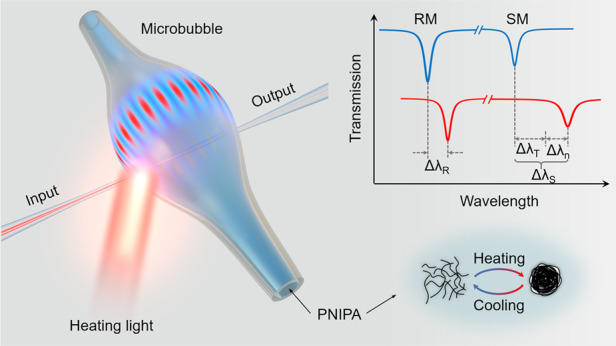

## Introduction

Micro-/nano-photonic devices have provided increasing opportunities for optical sensing and detection in the past decades, with intriguing features of miniature size, non-invasiveness and fast response, etc.^1–9^. In particular, high-*Q* microcavities manifest themselves by virtue of the strongly enhanced light-matter interaction, and have witnessed tremendous progress of ultrasensitive optical sensing^10–17^. For example, the detection limit of the microcavity sensors has reached levels of single molecules or single ions by employing mode-locking, optical spring, or plasmonic enhancement methods^18–21^. Despite their achievements of ultrahigh sensitivities, the conventional schemes of microcavity sensing, for example, by monitoring the shift of a single resonance mode^12,22^, usually cannot identify mixed effects. Thus, most current optical microsensors are restricted to monitoring a single chemical/physical measurand at a time^23–25^. To measure more complicated processes involving multi-physical quantities, new detection technology with straightforward signal deconvolution capability is required urgently.

In this work, we experimentally monitor the transition dynamics of poly(N-isopropylacrylamide) (PNIPA), a typical phase-change material, by using a high-*Q* optofluidic microcavity, in which the integrated microfluidic channel allows efficient coupling between the resonant optical field and the PNIPA molecules for operando detection. By developing a self-referencing strategy with dual-modes of the microcavity, the changes of temperature and refractive index of PNIPA during the phase transition are individually extracted from the cavity-resonance spectra. In real-time measurements, the refractive index of PNIPA demonstrating a hysteresis phenomenon in the cycles between hydrophilic and hydrophobic states is observed for the first time. In addition, under thermal-equilibrium conditions, it is found that the refractive index of PNIPA exhibits a classical Boltzmann distribution depending on the heating power and manifests the threshold of the phase transition. This strategy combines microcavity photonics with microfluidics and phase change materials, in which not only the basic properties of phase change materials are well characterized with dual-mode, self-referencing spectra, but also the functional photonic devices, such as optical switches^26^ and optical memories^27^, can be constructed.

## Results

**Fig. 1:**
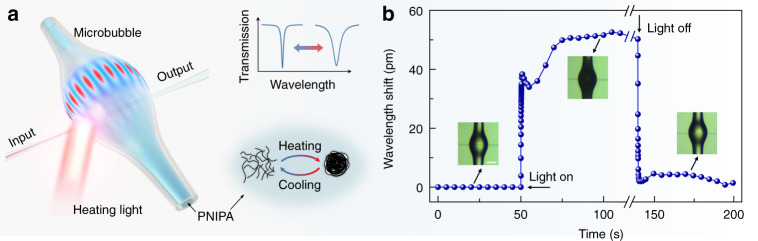
Optofluidic microcavity for PNIPA sensing. **a** Schematic of microbubble sensors for in operando monitoring of phase transition dynamics. Inset: the variation of WGM spectra correlated to the mesoscopic structures of PNIPA, where the cyclic phase transition changing between hydrophilic state and hydrophobic state. **b** Time-resolved wavelength shift of a typical WGM sensor during one cycle of heating-light on and off. Inset: Optical microscope images of PNIPA-microbubble at different stages, in which the bright (dark) image indicates hydrophilic (hydrophobic) state. The scale bar is 40 μm

PNIPA is a prototype thermo-responsive polymer and has drawn extensive attention in the applications of medicine, bioelectronics, optics, and robotics^28–32^. Below the lower critical solution temperature (LCST), the hydrophilic amide group of PNIPA is connected with water via hydrogen bond and exists as a fully hydrated random coil. As the temperature exceeds the LCST, PNIPA experiences a deswelling phase transition and transforms from the hydrophilic state to the hydrophobic state^33,34^ (Fig. 1a inset), mainly accompanied by a refractive index variation and heat transfer. In our experiment, 25% PNIPA solution (refractive index ~1.33) is prepared by polymerization of monomers (see Methods) and then injected into a silica hollow bubble microcavity (diameter ~80 μm) serving as a microfluidic channel for in operando monitoring the phase transition of PNIPA, after which the microbubble cavity is sealed using ultraviolet glue.

As shown in Fig. 1a, bubble microcavities support whispering-gallery modes (WGMs) via total internal reflection^35–37^. A tapered microfiber is used to evanescently couple the probe light at 780 nm (linewidth <200 kHz, power <0.11 mW) into the microcavity, and the transmission spectra of the resonant modes are measured, confirming *Q* factors over 10^6^ (see details in Supplementary Information). An external continuous infrared laser at 1550 nm is employed to locally heat the PNIPA solution and induce phase transition in a short time with a heating rate of 2–8 °C s^−1^ (Fig. S2 in Supplementary Information), resulted from the light absorption of the solution with an absorptivity of 7.667 cm^−1^^38^. Note that the pump light and probe light do not interfere with each other, because they are from two independent diode lasers, with different wavelengths and incoherent phases. The experiments are conducted in a cleanroom with the temperature of ~22 °C and the humidity of ~40%. As shown in Fig. 1b, during the cyclic phase transition process between the hydrophilic and hydrophobic states, resonance mode of the bubble microcavity experiences a continuous shift due to the change of both refractive index and temperature of PNIPA. Since the cavity resonances are encoded with the structural transition information of PNIPA, the WGM spectra exhibit nonmonotonic red-shift (blue-shift) in the heating (cooling) process. To verify the repeatability of our experimental setup, we carry out the experiment for over ten times with different microcavity devices at the same measurement condition, and the measurement results of each experiment are similar.

**Fig. 2:**
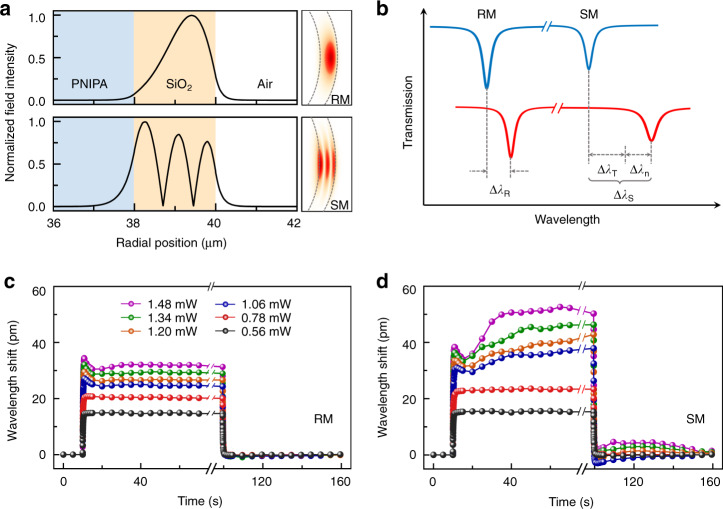
Concept of self-referencing WGM sensors. **a** Electrical field (|E|) distribution of reference mode (RM) and sensing mode (SM) by finite-element-method simulation. **b** Schematic of self-referencing strategy with dual-WGMs sensing method, where Δ*λ*_*T*_ = (*β*_2_*/β*_1_)⋅Δ*λ*_*R*_. **c**, **d** Real-time wavelength shifts of reference mode and sensing mode at different heating power and natural cooling (@ 22 °C), respectively

Here, the wavelength shift of the WGM sensor is simultaneously influenced by the thermo-optic effect and phase transition of PNIPA. Thus, it is a non-trivial task to resolve the contributions from each effect by solely tracing the spectrum of one WGM. To overcome this challenge, we develop a self-referencing sensing strategy to separately extract the two different contributions by simultaneously analyzing the shifts of a reference mode (RM) and a sensing mode (SM) which can be experimentally identified through mode broadening mechanism^39^. By further analyzing the field distribution in COMSOL simulation, it is calculated that over 99.94% optical field of the reference mode are confined in the solid wall of the silica bubble (shown in Fig. 2a, top), so that the resonance shift mainly arises from the absorption of the heating light and the subsequent refractive index change by the thermo-optic effect denoted as ∆*λ*_*R*_ (Fig. 2b). In contrast, there is a considerable part (~6.0%, two-orders of magnitude larger than that of the reference mode) of evanescent field penetrating into the PNIPA for the sensing mode (Fig. 2a, bottom), and the resonance shift ∆*λ*_*S*_ is caused by the thermo-optic effect ∆*λ*_*T*_ and intrinsic refractive index change ∆*λ*_*n*_ of PNIPA (Fig. 2b). Here the thermo-optic effect induced mode shifts of sensing and reference mode are usually different due to the distinct field distributions, but hold a constant ratio *β*_2_*/β*_1,_ which is derived from the experimental measurement. In this way, the mode shift by the intrinsic refractive index change of PNIPA during the phase transition is derived (see details in Methods):

1 $$\Delta \lambda _n = \Delta \lambda _S - \Delta \lambda _T = \Delta \lambda _S - \frac{{\beta _2}}{{\beta _1}}\Delta \lambda _R$$

Therefore, the temperature and refractive index of PNIPA during the phase transition can be deciphered by the dual-WGMs sensor.

Experimentally, we monitor the spectral shifts of both the reference mode and sensing mode in real time upon optical heating (response time ~0.4 s) and ambient cooling operations (response time ~1.0 s), as shown in Fig. 2c, d. Under the weak heating power (red and black curves), both the reference mode and sensing mode experience red shifts rapidly after the heating light is on within the time scale ~0.4 s, mainly arising from the thermo-optic effect. As seen, the sensing mode shifts a bit larger than reference mode with a ratio of *β*_2_*/β*_1_ ~1.03 because of the different field distributions. Then, the resonances of the two modes remain at the constant wavelengths, indicating that the temperature reaches an equilibrium state.

**Fig. 3:**
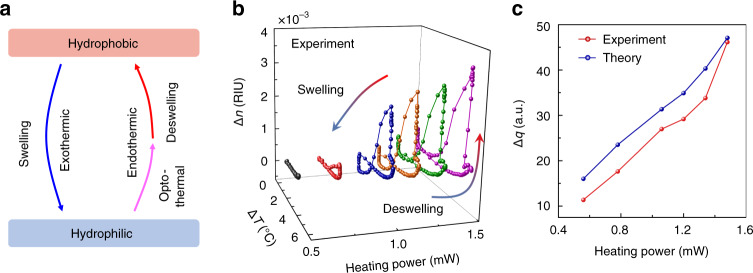
Probing the phase transition dynamics of PNIPA. **a** Diagram of phase transition process of PNIPA. The opto-thermal effect by infrared light increases the temperature of PNIPA solution, then it activates the de-swelling transition. The hydrophobic to hydrophilic transition is a direct swelling process by ambient cooling. **b** Dependence of the refractive index change on the temperature of PNIPA solution at different heating power in the experiment. **c** Dissipated heat (Δ*q*) in a transition cycle vs. the heating power by the experimental measurement (red curve) and theoretical calculation (blue curve)

With the further increase of the heating power, small blue-shifts emerge after the rapid red shift for both reference and sensing modes, since the deswelling transition absorbs the heat from the environment and results in the temperature decrease^40^. Then, during the deswelling transition process from the hydrophilic state to hydrophobic state, the reference mode reaches thermal equilibrium and stops shifting as time goes on, while, in contrast, the sensing mode keeps shifting towards long-wavelength. The aforementioned phenomena of the PNIPA solution under high heat power are absent in the case of microbubble filled with deionized water (Fig. S3c in Supplementary Information), indicating the exclusive effects of this phase transition dynamics. When the heating light is off, both the reference and sensing mode experience the fast blue-shift within the time scale ~1.0 s. Afterwards, the reference mode quickly reaches a stable state, while sensing mode exhibits slow red-shift and then gets its stable state which is probably due to the liquid convection inside the microbubble^41^. The physical processes of PNIPA phase transition triggered by illuminated infrared light are illustrated in Fig. 3a.

Furthermore, we separately extract the refractive index and temperature changes and reveal a clear hysteresis phenomenon during the PNIPA deswelling–swelling transition cycle. At each heating power, the PNIPA solution absorbs the light, and both the temperature and refractive index of the PNIPA solution change simultaneously, of which the dynamical evolution process is tracked and illustrated in the parametric space spanned by temperature and refractive index, as shown in Fig. 3b. In the heating–cooling cycle with the weak infrared light (for example, the black curve), the temperature increases and decreases reversibly, while the intrinsic refractive index keeps nearly unchanged, demonstrating that the phase transition does not happen at this stage. When the heating power is high enough (for example, the blue curve in Fig. 3b), the hydrophilic PNIPA absorbs energy under the heating operation, and the temperature rises until the emergence of the deswelling process. Subsequently, the temperature decreases by less than 0.5 °C, and meanwhile the refractive index of the PNIPA solution begins to increase, featuring the start of the deswelling transition. In the following process, the temperature remains constant, while the intrinsic refractive index increases monotonously, which is contrary to the initial process dominated by the thermo-optic effect. This phenomenon denotes the isothermal characteristics of the phase transition from the hydrophilic state to the hydrophobic state.

When the heating light is turned off, distinct from the deswelling process, the PNIPA solution experiences a swelling transition with the slow decline of intrinsic refractive index change relating to the temperature. Notably, this kind of irreversible transition forms a pronounced hysteresis loop of refractive index in a closed cycle, which is the typical behavior in phase transitions of responsive polymers^33,42,43^. Although the previously reported hysteresis was obtained from the measurement of hydrodynamic radius^34^, heat capacity^44^, and infrared absorption fingerprint^43^, rather than the refractive index, the hysteresis phenomena by different approaches are attributed to the similar mechanism, i.e., the formation of extra intra- and inter-chain hydrogen bonds as the PNIPA coils collapsed to globules^33^. Besides, it is also noticed that an additional hysteresis loop appears at the end of the swelling transition, probably due to the solution convection inside the microbubble. We note that this is the first report of the hysteresis effect in phase change materials by using the microcavity sensor, to the best of our knowledge. According to the theoretical analysis based on Flory-Rehner theory (see Methods and Fig. S5 in Supplementary Information), it is found that the encircled area of major hysteresis loop in the transition cycles corresponds to the dissipated heat in the cycle of structural transition, with respect to the irreversible fracture and formation of hydrogen bonds^34^. Besides, it is also found that the dissipated heat is positively associated with the heating power (shown in Fig. 3c), because the heat capacity of PNIPA is nearly proportional to the polymer volume fraction which is correlated to the heating power^44^.

**Fig. 4:**
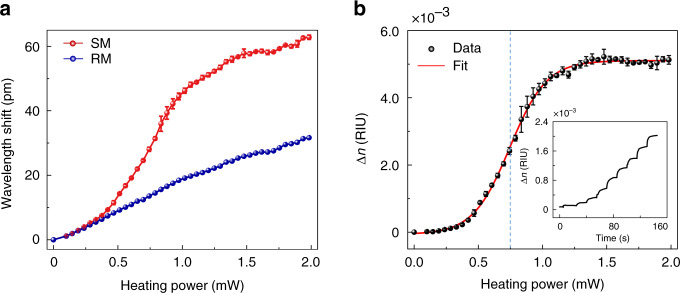
Steady-state phase transition dynamics. **a** Dependence of the wavelength shifts of reference mode and sensing mode on the heating light power. **b** Extracted refractive index change (∆*n*) of PNIPA solution on the heating light power. Inset: Extracted real-time refractive index change as the heating light power is stepwise increased

Besides the dynamic reaction of the phase transition, we also interrogate the steady-state response of PNIPA under the heating operation with different powers (Fig. 4). Experimentally, at each heating power, the eventual wavelength shifts of both the reference mode and sensing mode are recorded after about 20 s which is enough for thermal equilibrium, as shown in the inset of Fig. 4b. Note that here the required time for thermal equilibrium is different from that in the measurements in Fig. 2c–d (for example, over 50 s at 1.06 mW), because each measurement at a certain power in Fig. 4a begins with a bias-heating power. Under the weak heating light lower than 0.5 mW, both the sensing mode and reference mode experience linear wavelength shifts approximately, with a slope ratio of *β*_2_*/β*_1_. As the heating power further rises, the reference mode still shifts linearly, whereas the sensing mode significantly deviates from linear shifts due to the occurrence of the deswelling phase transition. The information of the intrinsic refractive index is further extracted through de-convolving its thermo-optic response, as plotted in Fig. 4b. The dependence of the refractive index on the heating power follows the classical Boltzmann distribution^45^, where the threshold power of the transition is calculated as 0.75 mW (blue dashed line). It is also found that the saturation of the refractive index emerges gradually with the heating power higher than 1.5 mW, indicating that all the PNIPA inside the microbubble transforms into the hydrophobic state.

## Discussion

In summary, we have experimentally investigated the phase-transition dynamics of the responsive polymer PNIPA by using an ultrahigh-*Q* microfluidic cavity. Benefitting from a self-referencing strategy employing dual-WGMs, the information of the refractive index and temperature of PNIPA during the phase transition is successfully decoded from the transmission spectra of the microcavity. Consequently, a hysteresis phenomenon of the refractive index change is observed in the cycle of deswelling–swelling transition, of which the encircled area corresponds to the dissipated heat. Additionally, the steady-state evolution is analyzed, exhibiting a Boltzmann distribution with a transition threshold of 0.75 mW.

Besides our present method by microcavities, some other techniques have also been developed for monitoring phase-transition dynamics of responsive polymers, including calorimetry^46^, rheology^47^, and light scattering^48^. Nevertheless, all the existing methods are hampered by low sensitivity and slow response, so that some slightly fast variations in phase transition process are challenging to be observed, for example, the weak endothermic process measured in the initial deswelling transition in Fig. 3b. On the contrast, the microcavity sensors proposed in this work not only possess the universal merits (e.g., miniature footprint and fast response) of micro-/nano-photonic devices, but also manifest a lot of unique shining points. For instance, the multimode property of the WGM microcavity enables the simultaneous monitoring of multi-physical features in a single platform, and the ultrahigh-*Q* resonance allows trace measurements with a tiny amount of analyte. Besides, the compatibility with microfluidic systems can support small-volume sample handling for performing automated functions. We would also point out that this work is a fundamental proof-of-principle study, which can be readily extended to probe the phase transition of other phase-change materials, such as GeSbTe^49–51^, SrTiO_3_^52^, VO_2_^53^, and provide opportunities for exploring novel dynamic biochemical processes such as protein denaturation.

## Materials and methods

### PNIPA linear polymer preparation

The poly(N-isopropylacrylamide)-based polymer (PNIPA) was prepared as follows: Firstly, 10 g of N-isopropylacrylamide (NIPA) monomer was dissolved in 50 mL benzene with 0.16 g AIBN added as an initiator. The polymerization was carried out in water bath at 65 °C for 10 h under N_2_ protection. After polymerization, the benzene was removed by evaporation. The resulting crude polymer was further dried and dissolved in a small amount of acetone and then was put into *n*-hexane drop by drop. After the filtration, it was dried under a vacuum environment at 30 °C for 48 h and the white solid of PNIPA was obtained. Finally, the PNIPA solution was successfully prepared by dissolving the solid in deionized water. The average molecular mass of 1.65 × 10^4^ g mol^−1^ was measured by Agilent 1100 series gel chromatograph.

### Fabrication of microbubble cavity

The microbubble cavity is fabricated by the following steps. First, the fused-silica capillary with an outer diameter of 140 μm and inner diameter of 100 μm was tapered to an outer diameter of 30 μm using a heat-and-pull method. Next, the counter-propagating CO_2_ laser beams were focused onto the internally pressurized capillary, where the focused region expanded and the wall became thinner. Finally, a microbubble cavity with a diameter of ~80 μm and a wall thickness of 2.0–2.5 μm was formed by controlling the heating parameters.

### Theoretical models of self-referencing WGM sensor

By using a self-reference sensing strategy, we can eliminate thermal response and then obtain the intrinsic refractive index change purely induced by PNIPA structural transition. The PNIPA refractive index change contributing to the reference mode is two orders of magnitude smaller than that of the sensing mode, so it is ignored in the calculation. The wavelength shifts of RM and SM are expressed as:

1 $$\Delta \lambda _{{\mathrm{RM}}} = \lambda _1\alpha _1\Delta T/n_{{\mathrm{eff}},1} = \beta _1\Delta T$$

2 $$\Delta \lambda _{{\mathrm{SM}}} = \lambda _2\alpha _2\Delta T/n_{{\mathrm{eff}},2} + \gamma \Delta n = \beta _2\Delta T + \gamma \Delta n$$

where Δ*λ* stands for wavelength shift, λ_*i*_ is the WGM resonance wavelength ~779.50 nm, Δ*T* denotes the temperature variation of microbubble resonator, *α*_1_ is the effective themo-optic coefficients (TOC) of silica, *β*_1_ = 5.92 pm °C^−1^ is the temperature sensitivity of the reference mode, *α*_2_ represents the weighted TOC of silica and PNIPA solution, *n*_eff*,i*_ is effective refractive index of WGM, *γ* describes the refractive index change solely induced by structural transition. According to Eqs. (1) and (2), the Δ*n* induced by the phase transition of PNIPA is derived:

3 $$\gamma \Delta n = \Delta \lambda _{{\mathrm{SM}}} - \frac{{\beta _2}}{{\beta _1}}\Delta \lambda _{{\mathrm{RM}}}$$

where *γ* = 6151.14 pm RIU^−1^, *β*_2_*/β*_1_ = 1.03. Here, *γ* is determined by the field distribution of sensing mode employing the finite element method in COMSOL. In the simulation, the light wavelength is set as 780 nm, the diameter and wall thickness of the microbubble cavity are 80 μm and 2.0 μm, respectively. The ratio of *β*_2_*/β*_1_ is directly extracted from experiments.

### Theoretical models of phase transition dynamics

The phase-transition dynamics of PNIPA is modeled based on Flory–Rehner theory, which describes the transition kinetics of PNIPA polymer by balancing the elastic and mixing contributions to the osmotic pressure within polymers^54^. The Flory parameter *χ*, describes the change of free energy when a solvent–solvent contact is replaced by a solvent–polymer contact, and is expressed as^55,56^:

4 $$\chi = \frac{1}{2} - A\left( {1 - \frac{\theta }{T}} \right)$$

5 $$\chi = \frac{1}{{\varphi ^2}}\left\{ {\eta \left[ {\frac{\varphi }{{2\varphi _0}} - \left( {\frac{\varphi }{{\varphi _0}}} \right)^{1/3}} \right] - \varphi - {\mathrm{ln}}\left( {1 - \varphi } \right)} \right\}$$

where *A* represents the entropy change, *θ* is the theta temperature depends on both entropic and enthalpic variations, *η* is a dimensionless factor relating to the state of PNIPA (*η* = 5.26 × 10^−4^ in the heating process and 1.75 × 10^−4^ in cooling process in this experiment), *φ* is the polymer volume fraction and *φ*_0_ is the polymer volume fraction at reference state (*φ*_0_ = 0.03 in the heating process and 0.01 in cooling process in this experiment), and the *φ* can be calculated by Eqs. (4) and (5). In addition, the intrinsic refractive index change can be obtained by ∆*n*(*T*) = *m*⋅∆*φ*(*T*), where *m* = 0.19^57^.

In addition, we extract the heat dissipation in the cycle of structural transition both experimentally and theoretically by introducing a parameter, i.e., specific heat capacity. Here, we assume that the specific heat capacity of PNIPA solution is the linear superposition of water and PNIPA polymer and the coefficients depend on the volume fraction of water and polymer, i.e.

6 $$C\left( T \right) = \left( {1 - \varphi \left( T \right)} \right)C_{{\mathrm{water}}} + \varphi \left( T \right)C_{{\mathrm{polymer}}}$$

wherein *C*_water_ = 4.2 J g^−1^ K^−1^ and *C*_polyme*r*_ = 4.93 J g^−1^ K^−1^ denotes the heat capacity of water and PNIPA polymer, respectively^46^. Thus, the heat dissipation under a close cycle of transition is calculated by Δ*q* = ∮*C*(*T*) d*T*.

## Supplementary information

Graphical abstract

Supplementary Information
