# Supplementary material for: Operando monitoring transition dynamics of responsive polymer using optofluidic microcavities
Source: Light Sci Appl. 2021 Jun 16;10:128. doi: 10.1038/s41377-021-00570-1 (PMC8209048; doi:10.1038/s41377-021-00570-1)
Supplement: Supplementary file 1 — Graphical abstract [file 41377_2021_570_MOESM1_ESM.docx]

**Graphical abstract**


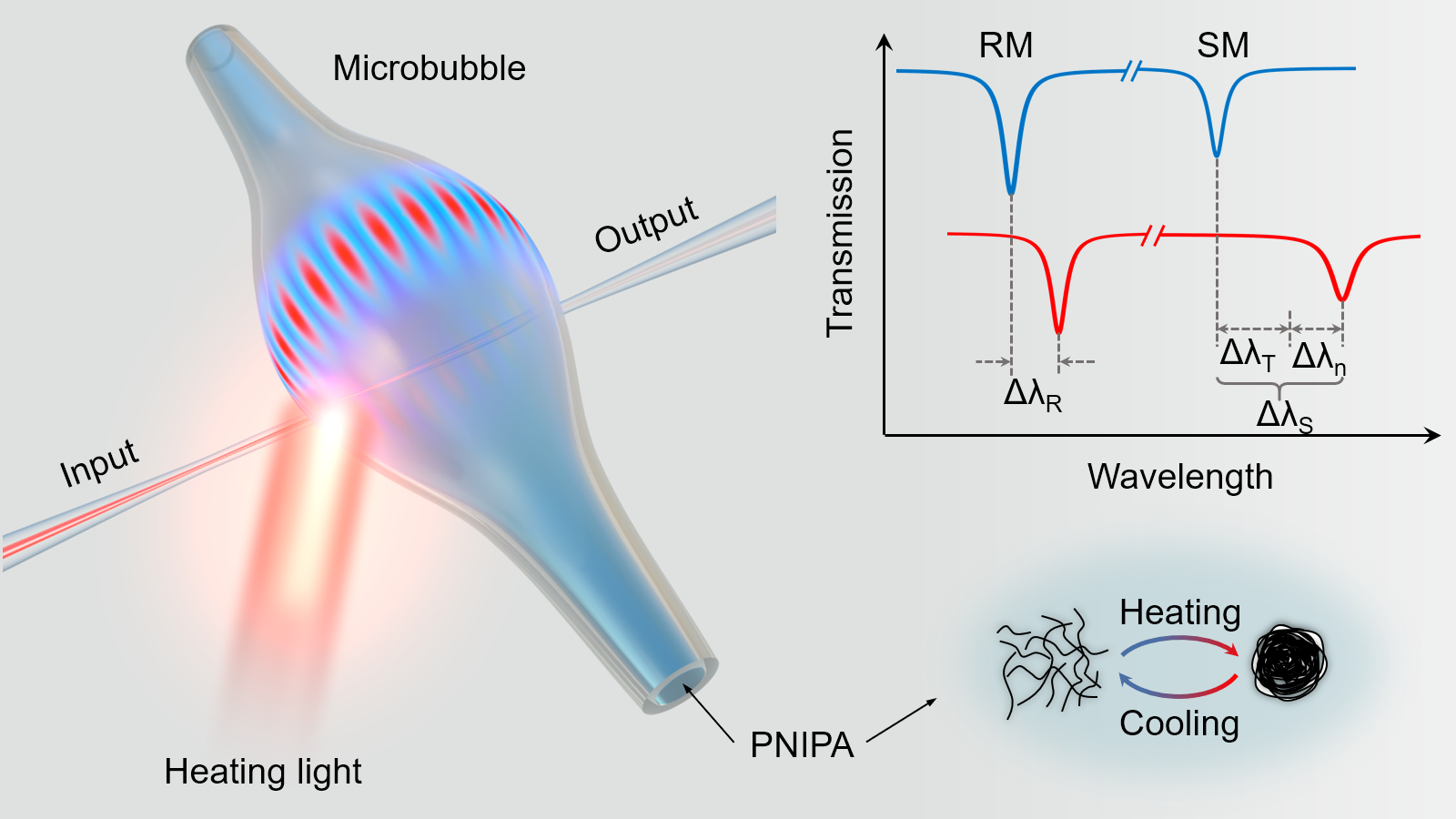


**Short summary:**

Operando monitoring transition dynamics of responsive polymer via a self-referencing optofluidic microcavity is demonstrated. The refractive index and temperature information of the analyte during the phase-transition process are precisely decoupled.
