# Supplementary material for: Operando monitoring transition dynamics of responsive polymer using optofluidic microcavities
Source: Light Sci Appl. 2021 Jun 16;10:128. doi: 10.1038/s41377-021-00570-1 (PMC8209048; doi:10.1038/s41377-021-00570-1)
Supplement: Supplementary file 2 — Supplementary Information [file 41377_2021_570_MOESM2_ESM.docx]

**Supplementary Information for “Operando monitoring transition dynamics of responsive polymer using optofluidic microcavities”**

Da-Quan Yang^1^^,†^, Jin-hui Chen^2,†^, Qi-Tao Cao^3,†^, Bing Duan^1^, Hao-Jing Chen^3^, Xiao-Chong Yu^4^, & Yun-Feng Xiao^3,5,6,^*

^1^School of Information and Communication Engineering, State Key Laboratory of Information Photonics and Optical Communications, Beijing University of Posts and Telecommunications, Beijing 100876, China

^2^Institute of Electromagnetics and Acoustics, Xiamen University, Xiamen 361005, China

^3^State Key Laboratory for Mesoscopic Physics and Frontiers Science Center for Nano-optoelectronics, School of Physics, Peking University, Beijing 100871, China

^4^Department of Physics and Applied Optics Beijing Area Major Laboratory, Beijing Normal University, Beijing 100875, China

^5^Collaborative Innovation Center of Extreme Optics, Shanxi University, Taiyuan 030006, China

^6^Peking University Yangtze Delta Institute of Optoelectronics, Nantong 226010, China

*Corresponding author: [yfxiao@pku.edu.cn](mailto:yfxiao@pku.edu.cn)

**ABSTRACT**

This Supplementary Information is organized as follows. In Section 1, we summarize the detailed experimental setup for the operando monitoring of phase transition dynamics. In Section 2, we provide the analysis and discussion of the heating rate. In Section 3, we summarize the wavelength shift of the resonant modes in the microbubble filled with PNIPA and water. In Section 4, we provide the mode intensity distribution of the microbubble cavity in the COMSOL simulation. In Section 5, we summarize the methods for probing the phase transition dynamics of PNIPA.

**Supplementary Note 1: Experimental setup**

The detailed experimental setup is shown in the Fig. S1. A tunable continuous wave laser at visible band (New Focus, TLB-6712) is coupled to the microbubble cavity via a tapered fiber to excite the target whispering gallery mode (WGM) for sensing. In detection, the WGM is characterized in the transmission spectrum with scanning frequency of the pump light. To accomplish this, the laser is tuned with a 50 Hz sine wave modulation signal from a function generator (Keysight, 33520B) for scanning the frequency. Then the transmitted light is collected by a low-noise photodetector (New Focus, 1801-FC) in real time and analyzed by a data acquisition system (NI, USB-6251). In the meantime, the microbubble is heated by an infrared laser at 1550 nm band (Toptica, CTL 1550) according with the absorption band of the poly (N-isopropylacrylamide) (PNIPA) solution.


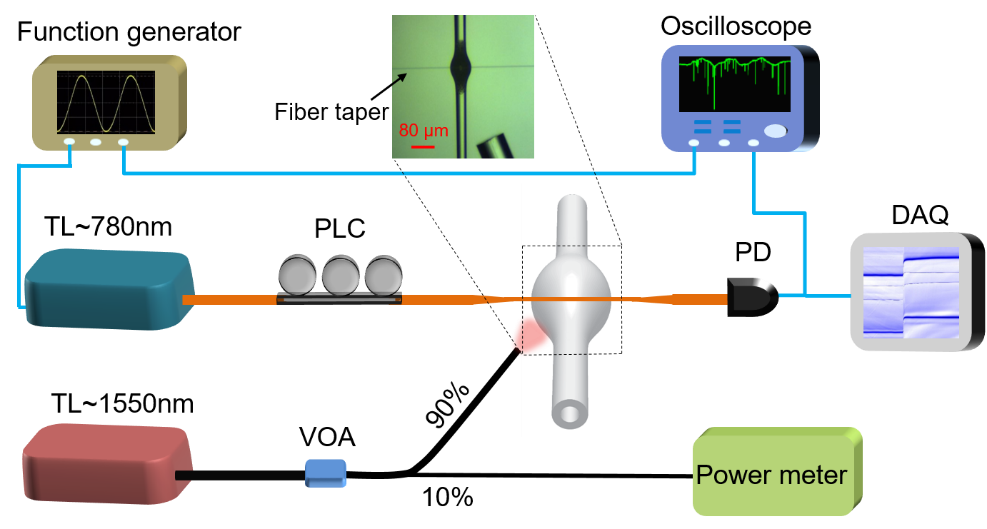


**Figure S1.** Schematic of experimental setup for in operando monitoring of phase transition dynamics. TL, tunable diode laser; PLC, polarization controller; VOA, variable optical attenuator; PD, photodetector; DAQ, data acquisition system.

**Supplementary Note 2: Heating rate**

The heating rate is confirmed according to the wavelength shift of the reference mode (RM). Here the heating rate is defined as the increased temperature of the PNIPA solution per second under a certain pump power. As shown in the Fig. S2, the heating rate is positively dependent on the heating power. It is found that the optical heating method used in this work has a high heating efficiency for that the light absorption and the temperature change is highly localized around the illuminating area (~ 2,000 μm^2^). Note that the measured maximum heating rate ~ 8 °C·s^-1^ is two-order of magnitude larger than that of the differential scanning calorimetry [1], thus the phase transition can be triggered rapidly.

As expected, the measured heating rate is nearly linearly related to the heating power when the heating optical power is smaller than ~ 1.1 mW. The deviation from linearity when the heating power is further increased is probably attributed to the phase change of PNIPA and its physical properties such as the thermal capacity [1], are altered accordingly. Further research is needed to fully understand this interesting phenomenon.


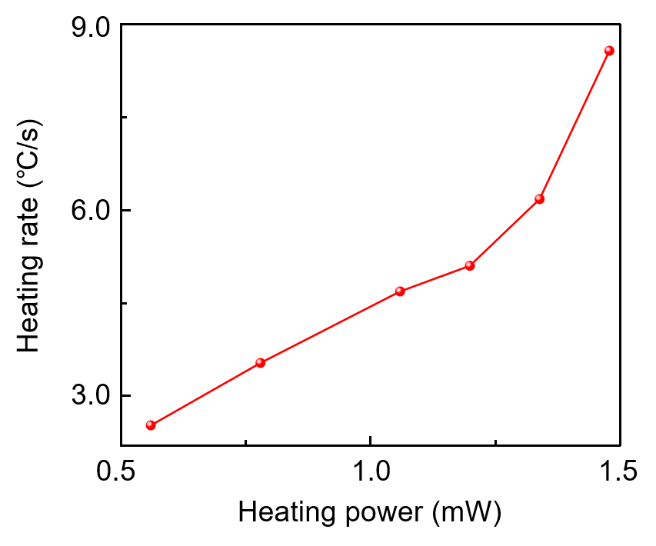


**Figure S2.** Dependence of heating rate with heating power. The heating rate is positively associated with the heating power.

**Supplementary Note 3: Resonant wavelength shift**

We monitor the transmission spectra evolution in real-time at the optical heating process when the microbubble is filled with PNIPA, as shown in Fig. S3a. The linewidth of the sensing mode (SM) is also broadened in the phase transition process because of the significantly enhanced light scattering. In experiment, the wavelength shift of the reference mode reaches a constant level within a few seconds after the light is on, while the sensing mode experiences a slow red-shift due to the PNIPA phase transition, as shown in Fig. S3b. As a comparison, when the microbubble is filled with deionized water, both of the reference mode and sensing mode experience a sharp red-shift before reaching a platform, as shown in Fig. S3c. It is noted that the wavelength shift of the sensing mode is explicitly smaller than that of the reference mode, since the field of sensing mode locates more inside the microbubble where the filled water exhibits a negative thermo-optic coefficient. The wavelength shift exhibits different time response when the bubble microcavity is filled with PNIPA and deionized water, which indicates the exclusive dynamics of PNIPA phase transition.


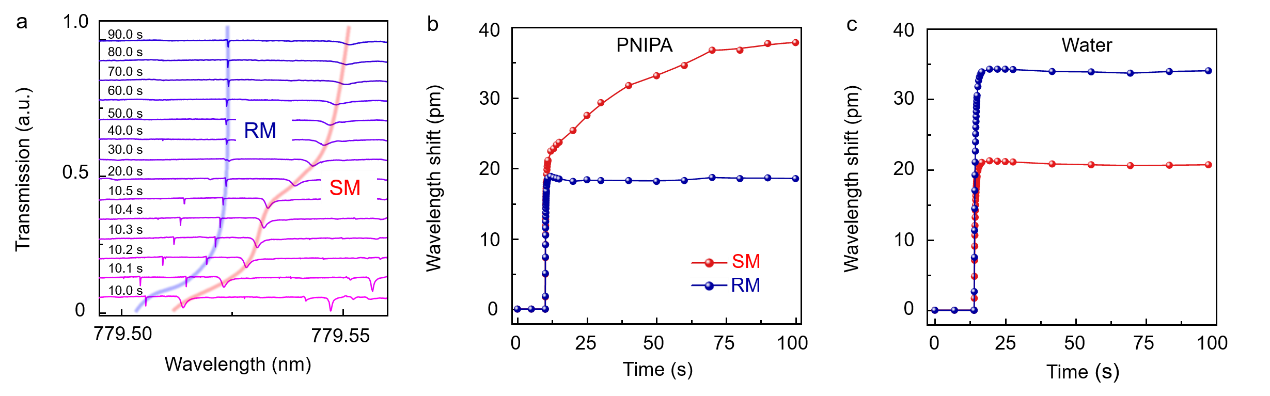


**Figure S3. a**, Real time transmission spectra evolution monitored during the phase transition process of PNIPA. The evolution from the bottom to top corresponds to the hydrophilic-hydrophobic transition. **b**, The extracted real-time WGM spectral response of PNIPA-bubble microcavity. The fast rising of wavelength shift is due to opto-thermal effect of infrared light illumination. The reference mode quickly reaches the thermal balance state, while the sensing mode experiences a slow red-shift by the structural transition of PNIPA. **c**, The extracted real-time WGM wavelength shifts of water-bubble microcavity. Both of the reference mode and sensing mode quickly reach a thermal-balance state with heat light.

**Supplementary Note 4: Field distribution of** **microbubble in simulation**

To model the geometry of the microbubble cavity in the simulation, a rotationally axisymmetric model is applied in the two-dimensional COMSOL simulation, as shown in Fig. S4. In the simulation model, the wavelength of light is approximately 780 nm; the diameter and the wall thickness of the microbubble cavity are 80 μm and 2.0 μm, respectively. The region 1, region 2 and region 3 are separately set as silica, PNIPA solution, and air, with the corresponding refractive indices *n_1_* = 1.45, *n_2_* = 1.33, *n_3_* = 1.0. There is a perfectly matched layer in the outermost rectangular region for infinite space simulation.


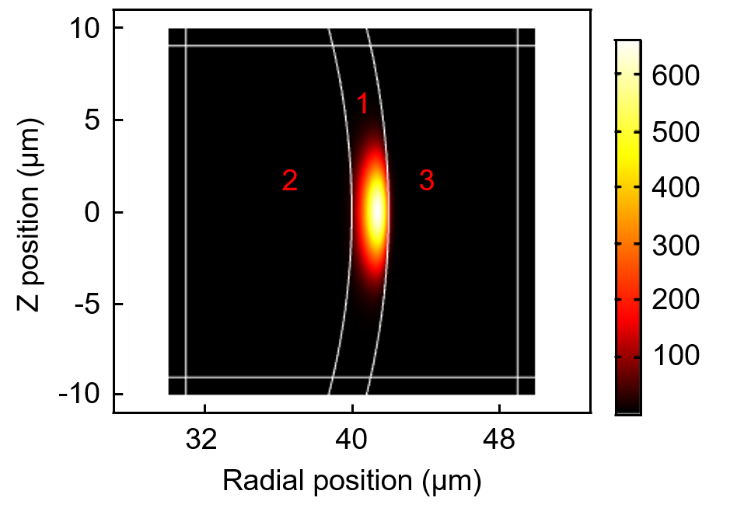


**Figure S4.** Schematic of a microbubble cavity model in COMSOL.

**Supplementary Note 5: Probing the phase transition dynamics of PNIPA**

We also build a theoretical model to describe the transition dynamics based on the Flory-Rehner theory. The calculated change of the refractive index under different heating power is plotted in Fig. S5, which is generally in accord with the experimental results. Nevertheless, it is noted that there are some inconsistences compared with the measurements, including the absences of the small temperature decrease before the deswelling transition and an additional hysteresis loop at the end of the swelling transition. The former is explained as the endothermic-reactions induced temperature decrease, and the latter is probably due to the solution convection inside the microbubble, which are not involved in the present theoretical mode.


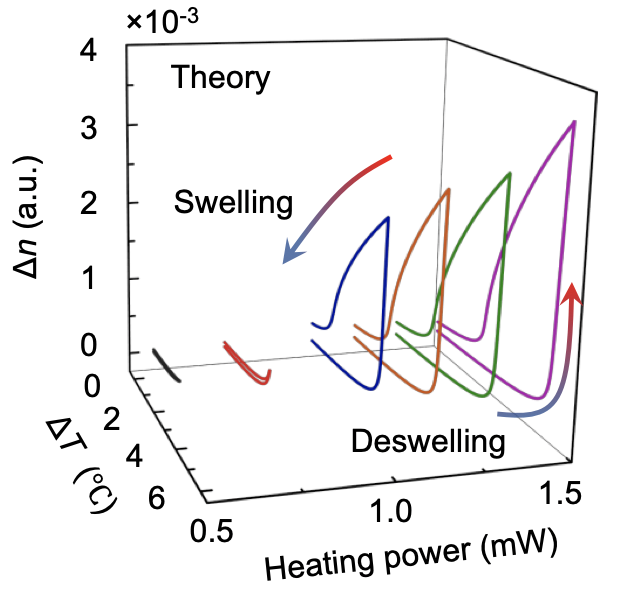


**Figure S5.** Dependence of the refractive index (RI) change on the temperature of PNIPA solution at different heating power in the theory.

**Reference:**

[1] Ding Y. W., Ye X. D., & Zhang G. Z. Microcalorimetric investigation on aggregation and dissolution of poly(N-isopropylacrylamide) chains in water, *Macromolecules* **38**, 904-908 (2005).
